# Information Security Scheme Based on Computational Temporal Ghost Imaging

**DOI:** 10.1038/s41598-017-07816-2

**Published:** 2017-08-09

**Authors:** Shan Jiang, Yurong Wang, Tao Long, Xiangfeng Meng, Xiulun Yang, Rong Shu, Baoqing Sun

**Affiliations:** 1 School of Information Science and Engineering, Shandong University; Shandong Provincial Key Laboratory of Laser Technology and Application, Jinan, 250100 China; 2 Key Laboratory of Space Active Opto-Electronics Technology of CAS, Shanghai Institute of Technical Physics, Chinese Academy of Sciences, Shanghai, 200083 China

## Abstract

An information security scheme based on computational temporal ghost imaging is proposed. A sequence of independent 2D random binary patterns are used as encryption key to multiply with the 1D data stream. The cipher text is obtained by summing the weighted encryption key. The decryption process can be realized by correlation measurement between the encrypted information and the encryption key. Due to the instinct high-level randomness of the key, the security of this method is greatly guaranteed. The feasibility of this method and robustness against both occlusion and additional noise attacks are discussed with simulation, respectively.

## Introduction

Information security is an important factor in modern communication and propagation techniques. Among those research fields of information security, optical information security has become increasingly popular in recent years. After double random phase encoding (DRPE) technique proposed by Refregier and Javidi in 1995^1^, many variant encryption techniques based on optics principles have been proposed and demonstrated, such as using Fourier transform^2^, the fractional Fourier transform^3^, the Fresnel transform^4^, the gyrator transform^5^, the joint transform correlator^6^, the interference principle^7^, digital holography^8^, diffractive imaging^9^, polarization encoding^10^, and so on. Ghost imaging (GI) is a technique that produces the image of an object by correlating the intensity of two light beams, neither of which independently carries information about the shape of the object^11, 12^. Computational ghost imaging (CGI)^13, 14^ as a computational version of GI uses programmable illumination and largely simplifies the implementation of GI. Some potential applications of CGI in 3D imaging^15^, remote sensing^16^, radar detection^17^, and image encryption^18, 19^ have also been studied. Compared with traditional optical information encryption, CGI provides a radically different and alternative approach for optical encryption. It has the advantages of both better security and robustness owing to the randomness of the special secret key and simple optical realization. However, strict requirement of synchronizing detection makes CGI hardly to realize ultrafast signal encryption.

Recently a novel technique called temporal ghost imaging (TGI), which transfers the concept of GI to the temporal domain by taking into account space–time duality in optics, has been investigated theoretically, numerically and demonstrated experimentally both in classical light state and bi-photon state^20–25^. In contrast with GI, which reconstructs the image of a spatial object, TGI can retrieve of a temporal signal with a novel correlation algorithm. More recently, computational temporal ghost imaging (CTGI)^26^ has also been proposed and demonstrated experimentally, which is inspired by CGI and can greatly simplify the setup of TGI and gain a better reconstruction of a single non-reproducible, periodic or non-periodic, temporal signal. In CTGI, the temporal signal reconstruction is performed by a single shot, spatially multiplexed, measurement of the spatial intensity correlations between computer-generated random images and the images modulated by the temporal signal, recorded and summed on a chip CMOS camera used with no temporal resolution.

In this paper, we propose a new scheme of information encryption based on the concepts of CTGI, which keeps the advantages of CGI and meanwhile overcomes its weakness due to the fact that CTGI can reconstruct ultrafast signal with a slow detector after a long exposure time^23^. The original information to be encrypted, which is usually termed plaintext, is transformed into one-dimensional (1D) digital data stream. The encryption/decryption key is a stack of independent, computer-generated, two-dimensional (2D) random binary patterns with a large number of pixels. A new 2D pattern is generated by multiplying each pattern of the stack with the corresponding element of the 1D data stream in sequence and summing all the weighted 2D random binary patterns. This rendered 2D pattern is utilized as the encrypted information, which is regarded as the cipher text. The decryption process can be realized by using correlation measurement or calculation between the encrypted information and the secret keys. In this process, the 1D data stream and the secret key are analogous to the temporal signal to be reconstructed and the randomly fluctuating transverse intensity patterns emitted from light source or spatial light modulator in CTGI, respectively. The summing process corresponds to the time integrated image with the camera during a long exposure time. The rest of the paper is organized as follows: the principle of CTGI and proposed method are described in detail in Method. In Result, a series of simulated experiments are provided to validate the feasibility and evaluate the performance of the proposed method.

## Results

**Figure 1:**
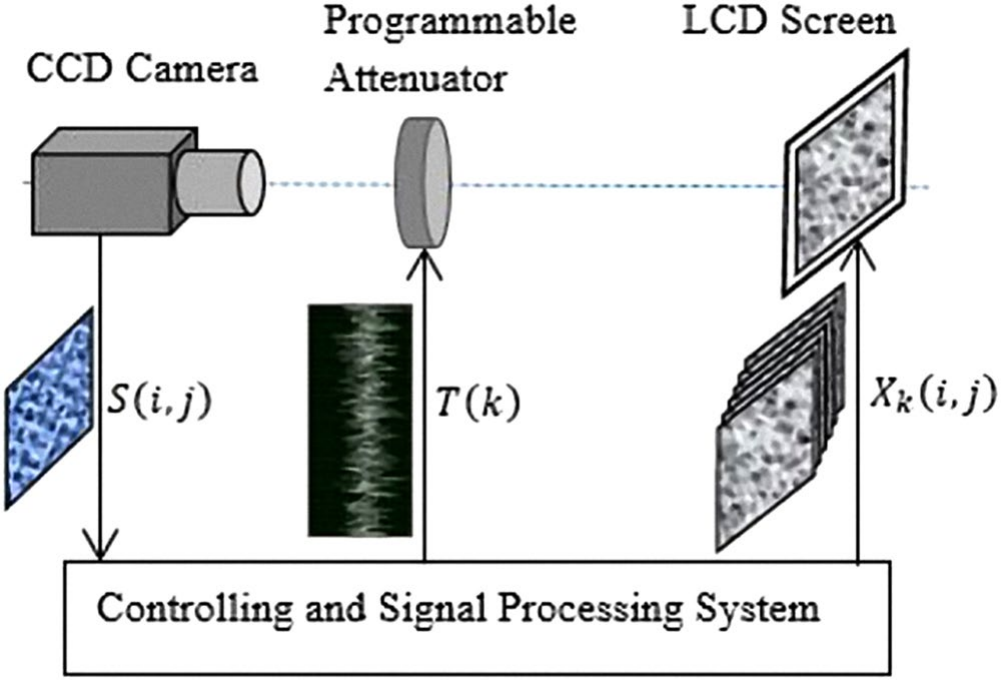
The schematic diagram of CTGI. Figure 1 is used to describe the principle and implementation of CTGI.

**Figure 2:**
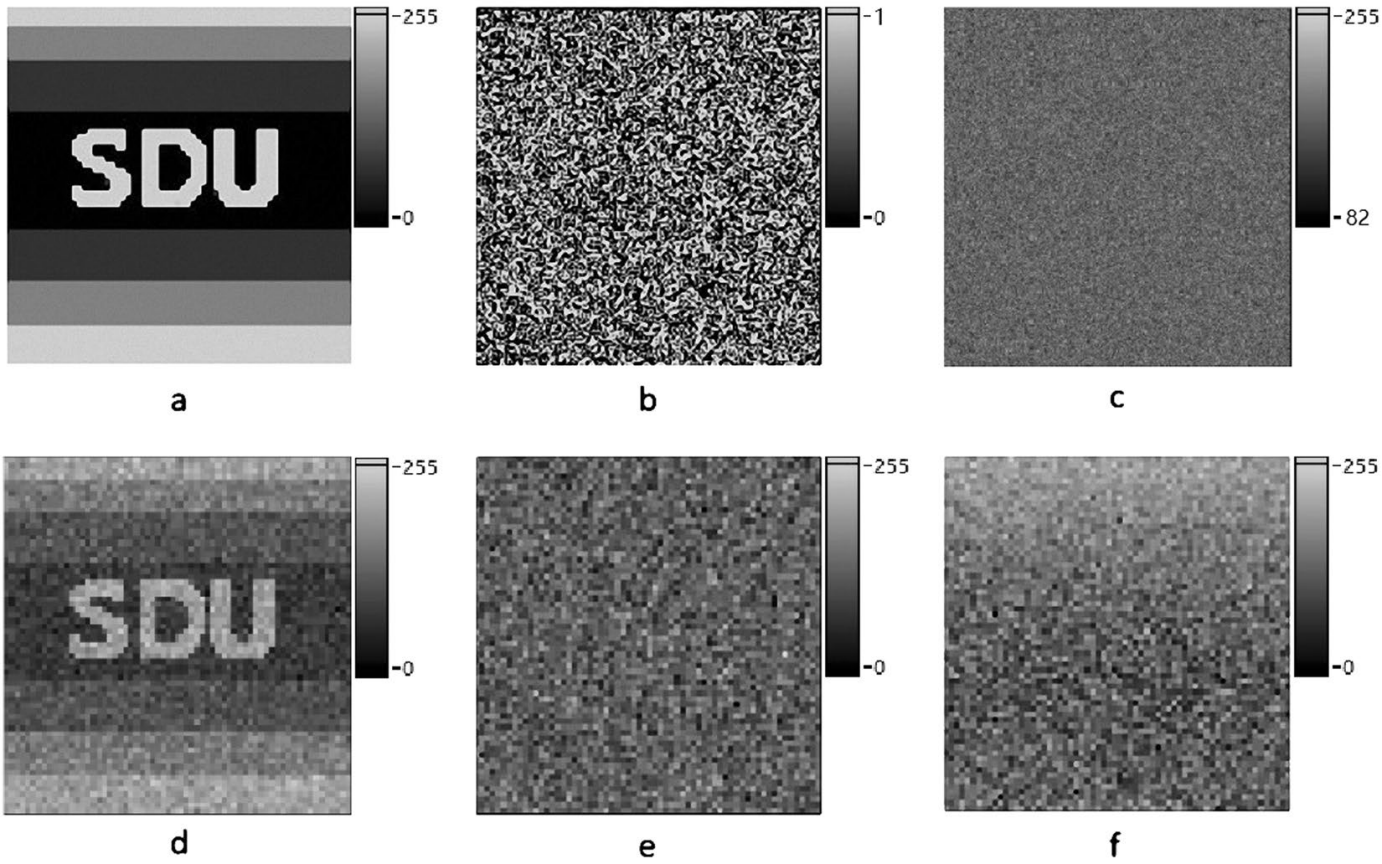
Experimental results of the proposed security scheme. (**a**) The original image to be encrypted. (**b**) One binary random pattern of the secret key sequence. (**c**) The image with a dynamic range of 8-bit grayscale after encrypted. (**d**) The reconstructed image with a dynamic range of 8-bit grayscale using correct key. (**e**) The decrypted image using the key with completely different spatial distribution. (**f**) The decrypted image using wrong order of the key.

A proof-of-principle simulation, which is based on the setup in Fig. 1, is employed to analyze the feasibility of this new encryption scheme. The setup includes a LCD screen, an attenuator, and a multi-pixel detector. In the encryption process, the LCD screen displays a sequence of random patterns working as the encryption key. Subsequently, these patterns travel through the attenuator and get their intensities modified uniformly depended on the attenuation. As the attenuation is controlled by a 1D weight signal sequence which is transformed from the 2D original image, each image obtained and superimposed in the multi-pixel detector is a modulated random encryption key, weighted by corresponding weight signal. In our simulation, the original image *F*_*O*_(*m*, *n*) is an 8-bits grayscale image in a resolution of 64 × 64-pixels, as shown in Fig. 2(a). The multi-pixel detector is set to a dynamic range of 8-bit in order to have a realistic simulation result. The original image is read as plaintext by computer and rearranged to a 1D array *T*_*i*_. The secret key *K*_*j*_(*p*, *q*), consists of 4096 independent 2D random binary patterns with 256 × 256 pixels. One of these secret keys is depicted in Fig. 2(b). The weighted sum pattern *F*′(*p*, *q*) is obtained and demonstrated in Fig. 2(c), which is the cipher text and can be stored locally or transmitted to the receiver. In the decryption stage, the receiver can utilize the same secret key sequence to decode the cipher text, the final decrypted image is displayed in Fig. 2(d). It should be emphasized that there is only one single shot for the encryption progress in this scheme, which potentially reduces the requirement for the response speed of detector. Therefore this scheme can be realized by using a very simple setup with low requirement of frame frequency of detector. All the equations (e.g. from Eq. (1) to Eq. (9)) and details about the encryption principle are defined in section Method.

**Figure 3:**
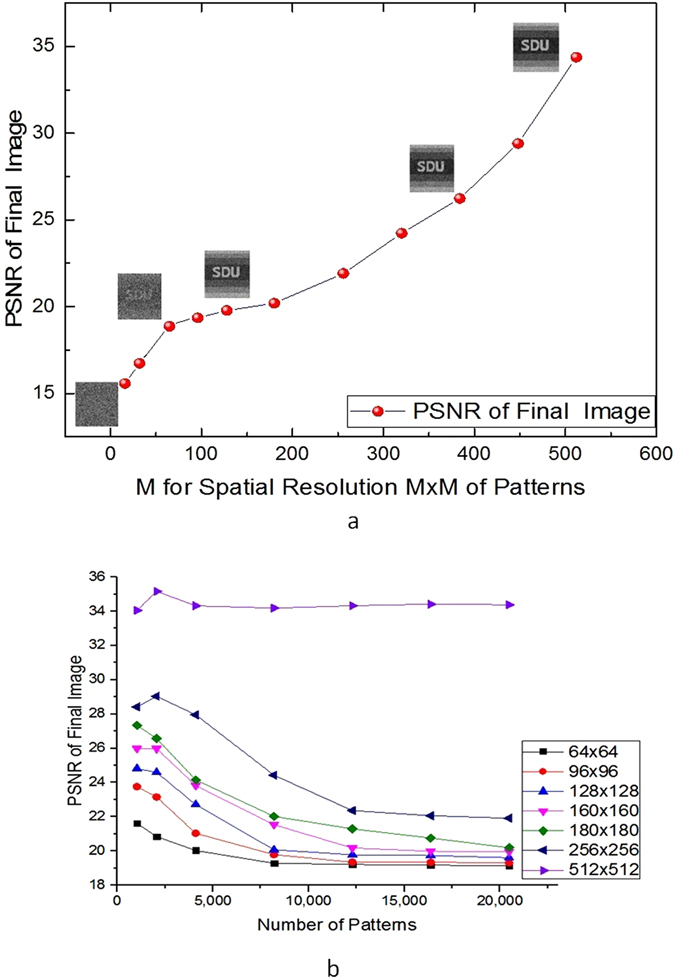
PSNR VS (**a**) spatial resolution of patterns and (**b**) number of patterns. (**a**) PSNR for the reconstructed image as a function of spatial resolution of the binary patterns in the secret key. (**b**) PSNR for the reconstructed image as a function of number of the binary patterns in the secret key.

To analyze the quality of the reconstructed image after decryption, the peak signal to noise ratio (PSNR) is utilized as the evaluation standard, which can be described as Equation (8) in section Method. The PSNR for the reconstructed image as a function of the spatial resolution (the pixel number *M* × *M*) of the binary pattern in the secret key is exhibited in Fig. 3(a). The number of pixels of random patterns denotes the number of measurements of each pixel in original image. When it is much larger than the pixel number of the original image, the number of measurements of the system becomes much larger than the pixel number of original image. It is clear that the PSNR develops by increasing spatial resolution of binary patterns of the secret key. For example, when the random pattern is 64 × 64-pixels, the PSNR of final image is 19.14 after 20480 times iteration, and as the size increases to 180 × 180-pixels and 512 × 512-pixels, the PSNR reaches 20.19 and 34.38 correspondingly. It can be deduced that the number of pixels of the random patterns should be adapted to the number of pixels of the original image and be not less than that of original image. As spatial resolution of random patterns is less than that of original image, PSNR of decipher text decreases obviously in Fig. 3(a). When spatial resolution of the random patterns is 64 × 64-pixels, PSNR of the reconstructed image is 18.25, which can be recognized but not very clear. However, if it comes to 32 × 32-pixels and 16 × 16-pixels, PSNR of the reconstructed image reduces to 16.72 and 15.57, respectively. Figure 3(a) proves that the number of pixels of random patterns should be larger than that of original image with the expectation of a proper result of decipher. Meanwhile, the number of patterns almost makes little contribution to PSNR of final image, when the number of measurements is not less than the number of pixels of the original image. Large numbers of patterns increase the impact of crosstalk, which decreases the PSNR of final image as shown in Fig. 3(b).

In order to check security of our proposed encryption scheme, two different kinds of wrong key sequences are introduced in decryption process. Figure 2(e) shows the decrypted image obtained after using the first kind of wrong key sequence. Compared with the right secret key sequence, the spatial distribution of each random patterns is completely distorted for the wrong key sequence. Figure 2(f) shows the decrypted image obtained after using the second kind of wrong key. Compared with the right secret key sequence, the only difference of the second kind of wrong key sequence is that the order of the patterns is disordered in random. It can be found that recovered images provide no valuable information about the plaintext image as expected. To analyze the security of the encryption system, the normalized root mean square (NRMS) is utilized as the evaluation standard, which is described as Equation (9) in section Method. The NRMS of the reconstructed images in Fig. 2(e) and (f) are 0.02 and 0.03, respectively. It is obviously declared that the correct decryption is based on an identical secret key sequence, both in spatial distribution and sort order.

**Figure 4:**
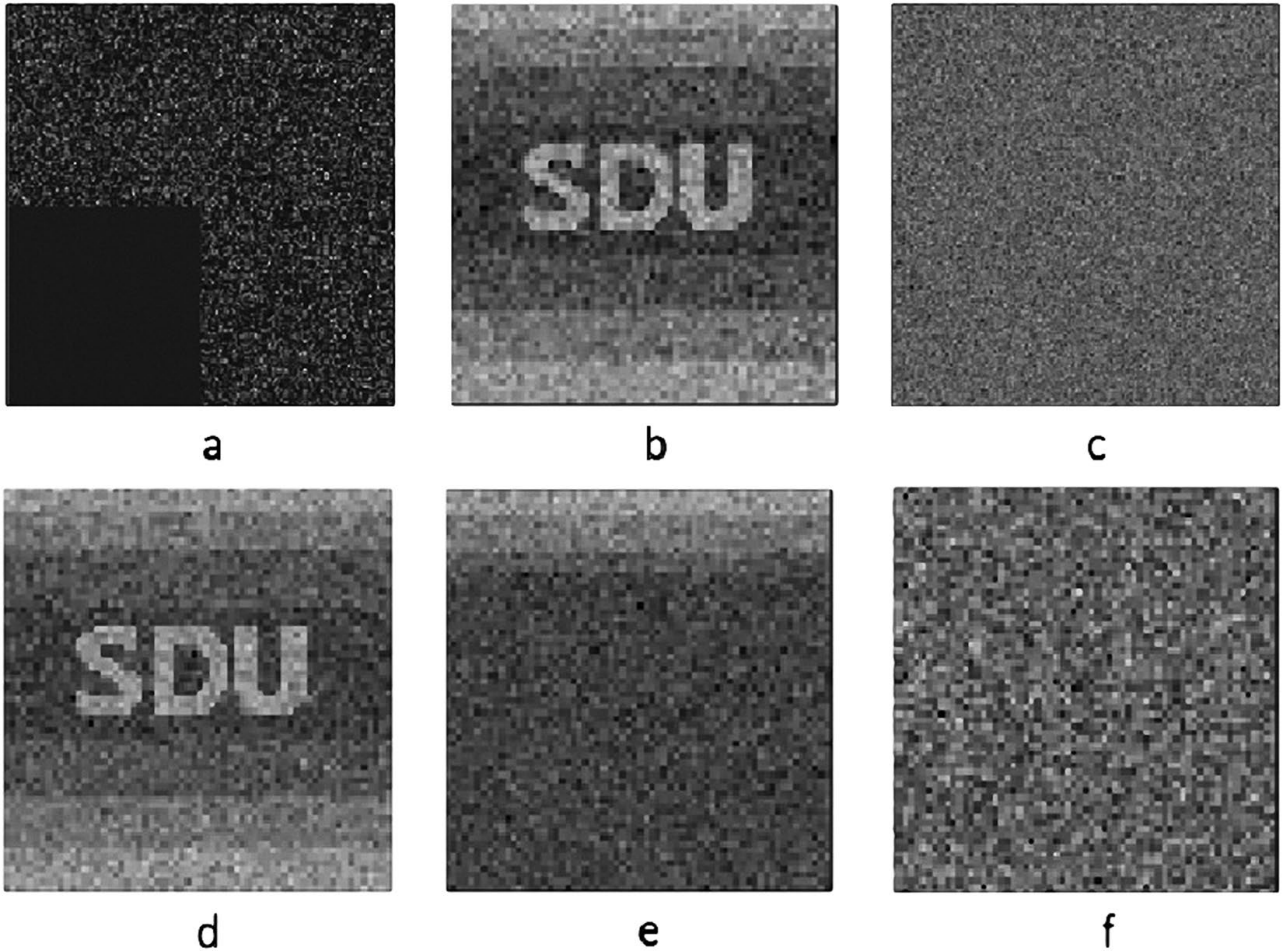
The result of robustness testing experiment. (**a**) The encrypted image with 25% occlusion. (**b**) The reconstructed image by the incomplete cipher text. (**c**) The cipher text after adding zero-mean white additive Gaussian noise. (**d**) The reconstructed image by the cipher text with noise. (**e**) The decrypted image using 20% patterns in the proper order. (**f**) The decrypted image using the right secret key sequence but without demodulation of cipher text when a modulation process on the cipher text is introduced.

**Figure 5:**
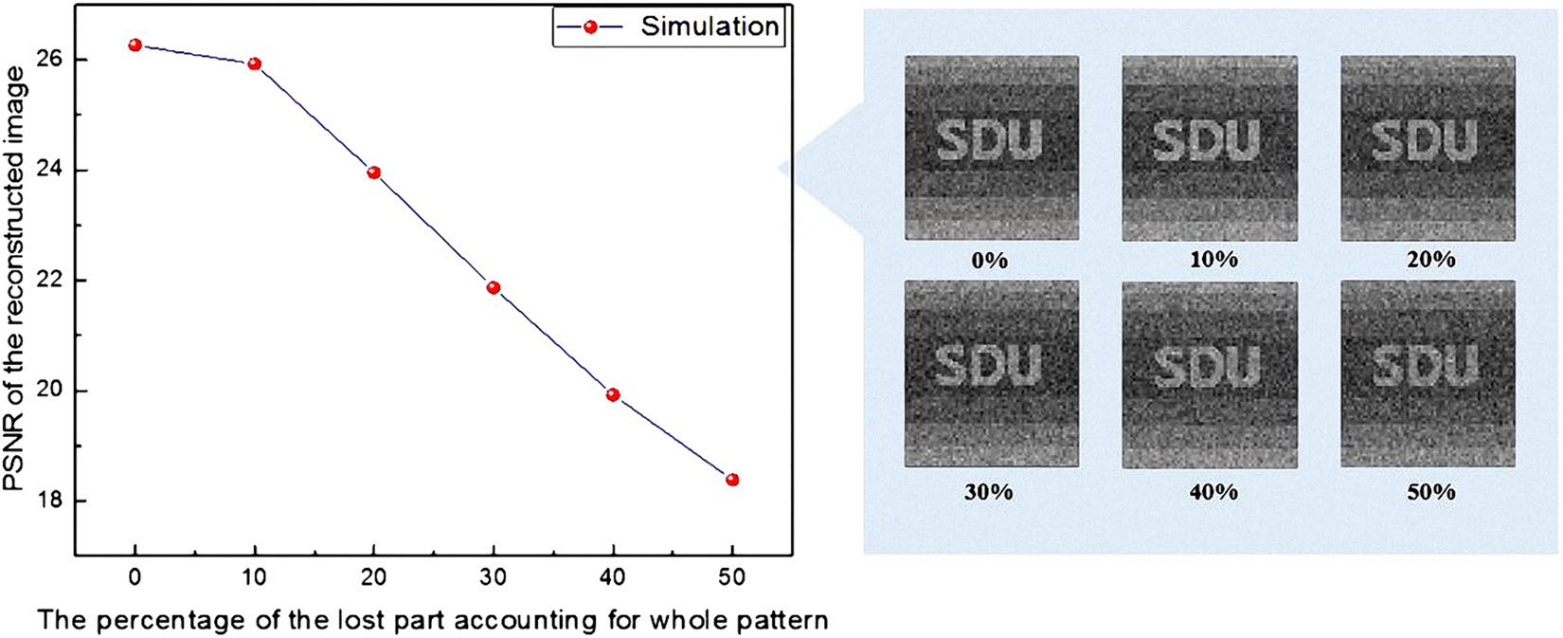
The PSNR for reconstructed image as a function of the percentage of the lost part accounting for the whole cipher text. Figure 5 shows the PSNR values of the retrieved images when the encrypted images are cropped by 10%, 20%, 30%, 40%, and 50%. The reconstructed image still have a good identification, although the PSNR decreases obviously.

The robustness of our proposed encryption system is tested by occlusion and noise attacks, which the cipher text is cropped by different percentage of the total number of pixels and distorted by white additive Gaussian noise. The encrypted image with 25% occlusion is depicted in Fig. 4(a), and the corresponding retrieved image with the PSNR of 22.99 is displayed in Fig. 4(b). Note that the values of the occluded pixels are replaced with 0 in our simulation as shown in Fig. 4(a). When the encrypted images are cropped by 10%, 20%, 30%, 40%, and 50%, the PSNR of the retrieved images are illustrated in Fig. 5. The PSNR of the reconstructed image reaches 18.38 even as the cipher text has only a half left. The number of remaining data of cipher text only effects the quality of reconstructed image but makes no contribution on data integrality of plaintext after decoding. Figure 4(c) and (d) demonstrate the encrypted image distorted by zero-mean white additive Gaussian noise with a standard deviation of 0.1 and corresponding retrieved image with the PSNR of 22.09.

The security of the scheme can be improved by adding extra encryption on the cipher text. As Fig. 4(e) shows, the information of corresponding pixel of original image can be reconstructed, if both small part (in this simulation is the first 20% of the total pixels) of the cipher text and the corresponding patterns of secret key sequence are achieved by eavesdropper. To avoid this problem, we choose to apply discrete cosine transformation (DCT) on the cipher text. And the decipher image can not be recognized directly without introducing the demodulation of cipher text, which is demonstrated in Fig. 4(f). It should be noted that this is just a simple example to increase the security. There is nothing that should stop the scheme safety to be further improved by adding more steps of encryption.

**Figure 6:**
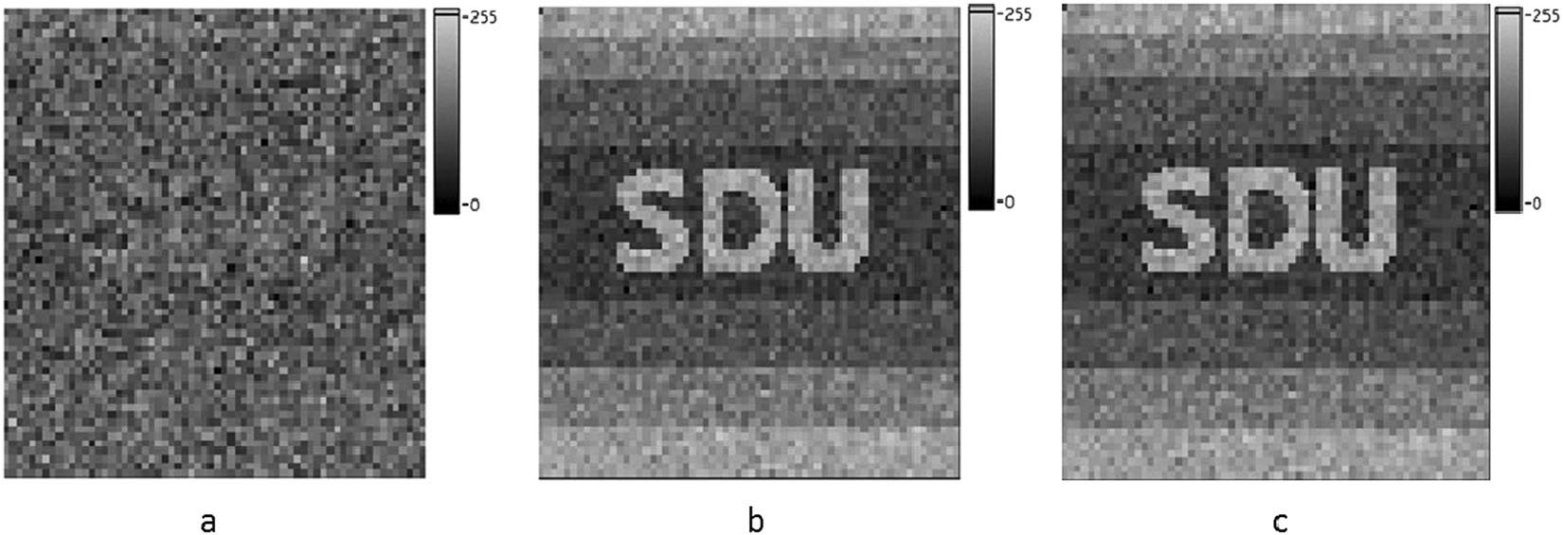
Results of reconstructed image with different dynamic ranges of detector. (**a**) Result of reconstructed image with the dynamic range of detector of 4-bit. (**b**) Result of reconstructed image with the dynamic range of detector of 8-bit. (**c**) Result of reconstructed image with the dynamic range of detector of 16-bit.

The influence of the dynamic range of detector is another important factor to be considered. Three different dynamic ranges of detector are set to 4-bits, 8-bits and 16-bits, respectively. Plain text in this simulation is a 8-bit grayscale pattern, which is the same with that in Fig. 2(a). After 4096 measurements, results are shown in Fig. 6. It is obvious that when the dynamic range of detector is smaller than the bit depth of plain text, the reconstruction is difficult to be recognized, which is exhibited in Fig. 6(a). However, with the increasing of dynamic range, the quality of reconstructed images becomes better, which is displayed in Fig. 6(b) and (c). The PSNR of them are 17.35, 27.03 and 27.86, respectively. Generally speaking, the dynamic range should be equal or larger than the bit depth of plain text. Otherwise, some of the information of plain text will be lost after an undersampled process. The simulated results in Figs 4, 5 and 6 demonstrate that the encryption scheme based on CTGI has a good performance in robustness.

The security can also be analyzed by probability analysis. According to Eq. (6), the value of reconstructed signal depends on both the spatial distribution and the order of secret key patterns. As for a binary pattern *K*_*j*_ in the secret key sequence with the size of *P* × *Q* pixels, the probability to construct or produce the same binary pattern, is 1⁄2(*P* × *Q*). When the secret key sequence consists of *J* binary patterns with the size of *P* × *Q*-pixels, the probability to construct or produce a same pattern sequence with the secret key is 1⁄(*J*! × 2(*P* × *Q* × *J*)). In most case, *J* is generally a large number, which means it is nearly impossible to generate a same secret key sequence without knowing the raw data stack. These results show that the proposed cryptosystem has immunity against blind decryption and also offers high level of security.

To summarize, a novel information security scheme based on computational temporal ghost imaging (CTGI) is proposed and evaluated in details in this paper. The original information is transformed into 1D digital data stream. The secret key, a stack of 2D random binary patterns, is multiplied with the corresponding element of the 1D data stream in sequence and then summed to generate a new pattern, which is the cipher text. The decryption process is realized by using correlation measurement or calculation between the encrypted information and the secret key. With this scheme, it becomes possible to encrypt ultrafast signal with quite low requirement of frame frequency of devices. This new proposal opens a new field of application for CTGI. Due to the complexity and randomness of the secret key in both spatial distribution and sequence-ordering, the scheme security is intrinsically guaranteed in an extreme high level. In principle, the encryption procedure of the proposed security scheme can not only be done computationally without using any optical devices, but also be implemented with practical optical system. In this paper, the feasibility of this method and its robustness against occlusion and additional noise attacks are verified by simulation. More systematical and comprehensive comparison between information security schemes based on CGI and CTGI and some other mainstream security scheme will be studied in our further work.

## Methods

### Computational Temporal Ghost Imaging

In ref. 26, Fabrice Devaux *et al*. introduced in detail the computational temporal ghost imaging (CTGI), which allows the retrieval of a single non-reproducible, periodic, or non-periodic temporal signal. Figure 1 is used to describe the principle and implementation of CTGI.

First, a stack of *K* independent random binary patterns *X*, with the size of *M* × *N* pixels, are computer-generated previously and then displayed sequentially on a liquid crystal display (LCD) screen. A programmable attenuator driven by the tested temporal signal *T*(*t*) (0 *≤* *T*(*t*) *≤* 1) is placed in the imaging system between the LCD screen and the CCD or CMOS camera. The time-varying transmission coefficient of the programmable attenuator is linearly determined by the temporal signal. These successively displayed binary patterns, which are weighted with the time-varying transmission coefficient, are imaged and summed on the CCD or CMOS camera during a long exposure time. In that case, the recorded image *S* corresponds to the time integrated image of the displayed patterns such that the level of a pixel *S*_*ij*_ of coordinates (*i*, *j*) is given by

1 $${{\rm{S}}}_{ij}={\sum }_{k=1}^{K}T(k){X}_{k}(i,j)$$

where *1* ≤ *i* ≤ *M* and *1* ≤ *j* ≤ *N*, and *T*(*k*), corresponding to the temporal signal, is the value of the transmission coefficient at the time when the *k-th* pattern is displayed with *1* ≤ *k* ≤ *K*. In the setup described in Ref. [26], according to the polarization of the light emitted by the LCD screen the programmable attenuator consists of a liquid crystal variable wave plate and linear polarizer in order to realize the linear modulation of the energy transmission by a temporal signal during the exposure time of the camera. Obviously, some other alternative methods may be used to construct the programmable attenuator according to the working principle of different light source or display device.

Then, the temporal signal to be tested can be reconstructed by calculating the intensity correlations between the time integrated image *S* and the images *X*′ of the *K* random patterns with the following equation

2 $$T\text{'}(k)=\frac{{\sum }_{j=1}^{N}{\sum }_{i=1}^{M}({S}_{ij}-\langle S\rangle )({X}_{k}^{^{\prime} }(i,j)-\langle {X}_{k}^{^{\prime} }(i,j)\rangle )}{{\sum }_{j=1}^{N}{\sum }_{i=1}^{M}{({X}_{k}^{^{\prime} }(i,j)-\langle {X}_{k}^{^{\prime} }(i,j)\rangle )}^{2}}$$

where *T*′(*k*) is the value of the reconstructed temporal signal at the “time” *k* when the pattern *X*_*k*_(*i*, *j*) with the index *k* in the binary pattern stack *X* is displayed, <•> is the operation of taking average. From Eq. (2) we can come to a conclusion that the value of the reconstructed signal depends on the spatial distribution of *S*_*ij*_ and *X*_*k*_′(*i*, *j*), and the index of the reconstructed signal only depends on the index of the binary modulation images *X*_*k*_′(*i*, *j*) in the sequence. Without knowing either the spatial distribution of both the time integrated pattern and the binary random patterns or the order, it is impossible to reconstruct the temporal signal correctly. This characteristic of CTGI offers great promise for image encryption or decryption.

### Proposed information security scheme

**Figure 7:**
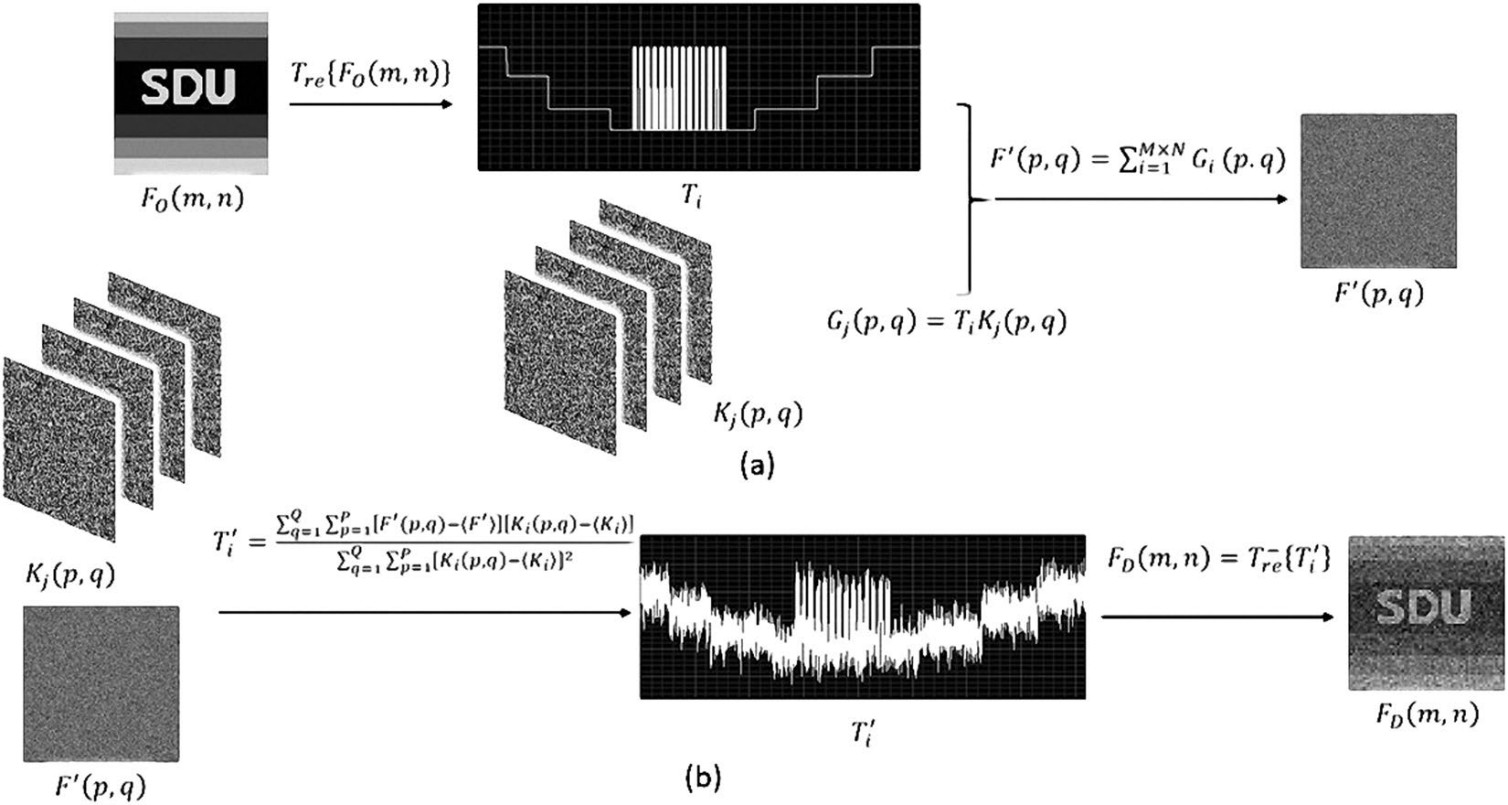
Algorithmic framework of the new encryption scheme based on CTGI. (**a**) The encryption procedure. (**b**) The decryption procedure.

The strategy of our proposed information security scheme is based on CTGI. With image encryption and decryption as an example, the schematic diagram and operation progress are shown in Fig. 7 and described in the following.

Figure 7(a) illustrates the encryption procedure. First, the original 2D image to be encrypted, *F*_*O*_(*m*, *n*) with size of *M* × *N* pixels, is rearranged into a 1D array *T*_*i*_ according to the following equation

3 $${T}_{i}={T}_{re}\{{F}_{o}(m,n)\}$$

where *m* = 1, 2, …, *M* and *n* = 1, 2, …, *N*. The operation *T*_*re*_{*∙*} denotes the 2D-1D rearrangement transformation, and in order to improve the security further, it may adopt some pixel scrambling techniques^27–29^. The subscript *i* = 1, 2, …, *M* × *N* is the number of the elements in the 1D array *T*_*i*_. A stack of *J* independent 2D random binary patterns, *K*_*j*_(*p*, *q*) with the size of *P* × *Q* pixels, has been previously generated and is used as the secret key with *p* = 1, 2, … *P*, *q* = 1, 2, … *Q*, *j* = 1, 2, … *J*, and *J* ≥ *M* × *N*. Here, the 1D array *T*_*i*_ and the secret key *K*_*j*_(*p*, *q*) correspond to the temporal signal *T*(*k*) and the random binary patterns *X*_*k*_ used in CTGI as shown in Eq. (1), respectively. Then, each element of the 1D array *T*_*i*_ interacts sequentially with each pattern *K*_*j*_(*p*, *q*) of the secret key, this process can be depicted with the following equation

4 $${G}_{j}(p,q)={T}_{i}{K}_{j}(p,q)$$

The operation represented in Eq. (4) is analogy to the modulation imposed by the time signal to the 2D random binary patterns in CTGI. In principle, in order to obtain a better security result, each element of the 1D array *T*_*i*_ can be interacted with several binary patterns, however, it will enlarge the length of the secret key sequence. So in the present analysis and the next experiments it is assumed that *J* = *M* × *N* and *j* = *i* in Eq. (4). Finally, analogy to the time integrated imaging process with the long exposure time detector in CTGI, the encrypted image can be obtained by summing the modulated secret key with the equation

5 $$F^{\prime} (p,q)=\sum _{j=1}^{M\times N}{G}_{j}(p,q)$$

The encrypted image *F*′(*p*, *q*), which is regarded as the cipher text, can be stored or transmitted without worrying about disclosure of information because the original image (the plaintext) has become a weighted value of each binary pattern of the secret key stack in the summing process. The above encryption process can be done in the computer without using any optical device. On the other hand, it can also be implemented in practical optical system with the help of a programmable attenuator controlled by a digital signal generator.

The decryption procedure is illustrated in Fig. 7(b) with assuming that the receiver shares the same stack of the secret key as the transmitter. First, analogy to the reconstruction process of the time signal in CTGI, a 1D array *T*_*i*_′ can be generated with the following equation

6 $${T}_{i}^{^{\prime} }=\frac{{\sum }_{q=1}^{Q}{\sum }_{p=1}^{P}[F^{\prime} (p,q)-F^{\prime} ][{K}_{i}(p,q)-{K}_{i}]}{{\sum }_{q=1}^{Q}{\sum }_{p=1}^{P}{[{K}_{i}(p,q)-{K}_{i}]}^{2}}$$

where *F*′ and *K*_*i*_ are the value of *F*′(*p*, *q*) and *K*_*i*_(*p*, *q*) after taking average, respectively. Then, the decrypted image *F*_*D*_(*m*, *n*) can be obtained by taking the inverse operation of the Eq. (1)

7 $${F}_{D}(m,n)={T}_{re}^{-}\{{T}_{i}^{^{\prime} }\}$$

where the operator ${T}_{re}^{-}${∙} denotes the 1D–2D rearrangement transform.

### Peak signal to noise ratio

The peak signal to noise ratio (PSNR), which is used to quantitatively analyze the quality of the reconstructed image after decrypting, is defined below,

8 $$PSNR=10{\mathrm{log}}_{10}\frac{1}{\frac{{\sum }_{n=1}^{N}{\sum }_{m=1}^{M}{({F}_{o}(m,n)-{F}_{D}(m,n))}^{2}}{M\times N}}$$

where *M* = *N* = 64, *F*_*o*_(*m*, *n*) and *F*_*D*_(*m*, *n*) denote the original image and the reconstructed image, respectively.

### Normalized root mean square

The normalized root mean square (NRMS), which are used to quantitatively analyze the security of the scheme, is defined below,

9 $${\rm{NRMS}}=\frac{\sqrt{{\sum }_{n=1}^{N}{\sum }_{m=1}^{M}{({F}_{o}(m,n)-{F}_{D}(m,n))}^{2}}}{\sqrt{{\sum }_{n=1}^{N}{\sum }_{m=1}^{M}{({F}_{o}(m,n))}^{2}}}$$

where *M* = *N* = 64, *F*_*o*_(*m*, *n*) and *F*_*D*_(*m*, *n*) denote the original image and the reconstructed image, respectively. The range of NRMS is from 0 to 1, which 0 denotes the highest security and 1 denotes the lowest security.
